# Collaborating with the Enemy: Function of Macrophages in the Development of Neoplastic Disease

**DOI:** 10.1155/2013/831387

**Published:** 2013-03-17

**Authors:** Andrzej Eljaszewicz, Małgorzata Wiese, Anna Helmin-Basa, Michal Jankowski, Lidia Gackowska, Izabela Kubiszewska, Wojciech Kaszewski, Jacek Michalkiewicz, Wojciech Zegarski

**Affiliations:** ^1^Chair of Immunology, Collegium Medicum in Bydgoszcz, Nicolaus Copernicus University of Torun, ul. M. Sklodowskiej-Curie 9, 85-094 Bydgoszcz, Poland; ^2^Department of Surgical Oncology, Collegium Medicum in Bydgoszcz, Nicolaus Copernicus University of Torun, ul. Romanowskiej 2, 85-796 Bydgoszcz, Poland; ^3^Department of Surgical Oncology, Prof. F. Łukaszczyk Memorial Center of Oncology in Bydgoszcz, ul. Romanowskiej 2, 85-796 Bydgoszcz, Poland; ^4^Department of Microbiology and Immunology, Children's Memorial Hospital, ul. Aleja Dzieci Polskich 20, 04-001 Warsaw, Poland

## Abstract

Due to the profile of released mediators (such as cytokines, chemokines, growth factors, etc.), neoplastic cells modulate the activity of immune system, directly affecting its components both locally and peripherally. This is reflected by the limited antineoplastic activity of the immune system (immunosuppressive effect), induction of tolerance to neoplastic antigens, and the promotion of processes associated with the proliferation of neoplastic tissue. Most of these responses are macrophages dependent, since these cells show proangiogenic properties, attenuate the adaptive response (anergization of naïve T lymphocytes, induction of Treg cell formation, polarization of immune response towards Th2, etc.), and support invasion and metastases formation. Tumor-associated macrophages (TAMs), a predominant component of leukocytic infiltrate, “cooperate” with the neoplastic tissue, leading to the intensified proliferation and the immune escape of the latter. This paper characterizes the function of macrophages in the development of neoplastic disease.

## 1. Introduction

Human body is exposed to a continuous influence of carcinogenic factors (physical, chemical, and biological), representing one of the reasons for the development of genetic mutations. Cells possess an array of mechanisms able to prevent mutations, as well as to repair DNA defects and eliminate genetically altered cells, for example, by the means of apoptosis [1, 2]. Disorders of this complicated protective system lead to the development of neoplastic cells, which, in turn, may be eliminated by an array of immunological mechanisms, including those affected by the innate immune system (monocytes, macrophages, NK cells, cytokines, etc.) and the adaptive immunity (induction of T and B lymphocytes). In order to eliminate neoplastic cells, the cells of the immune system must recognize them as “foreign.” The principles of recognition and the mechanisms of the immunological response are similar to those induced by foreign (bacterial, viral) or own antigens (autoantigens). Foreign antigens are highly immunogenic; that is, they induce immune response aimed at the rapid elimination of the infectious factors. These processes lead to the selection of a pool of immunocompetent cells, specialized in the destruction of a given factor. As previously mentioned, neoplastic cells originate from genetically altered cells of own tissues and therefore contain components that induce various degrees of immune tolerance, protecting them against the elimination by immunological mechanisms. On the other hand, neoplastic cells are characterized by a genetic instability [3], which manifests by changes in antigenic profile, which are not observed in the normal tissue. In some cases, this is accompanied by the overexpression of genes that remain inactive or exhibit low-level activity under normal conditions; many of the factors produced in this manner act as immunomodulators. It is widely known that neoplastic tissue can directly modulate its growth environment as a result of the activity of secreted cytokines and chemokines. This may be due to the following:

- chemotactic effect on leukocytes, including monocytes and macrophages;
- suppression of the activity of the immune system;
- regulation of neovascularization processes [4].

## 2. Chemotactic Factors for Monocytes/Macrophages Released by Neoplastic Cells

Some factors synthesized and released by neoplastic cells can induce leukocyte chemotaxis, including peripheral monocytes and macrophages located in the surrounding tissues. These cells represent the predominant component of leukocytic infiltrate of many tumors and, due to their pleotropic biological activity control, the majority of immunological processes proceeding in the region of neoplastic growth. Chemotaxis of monocytes and macrophages is a receptor-dependent process [5], directly associated with the polarization of these cells towards pro- or anti-inflammatory cells.

Monocyte chemotactic protein-1 (MCP-1/CCL2), showing affinity to CCR2 receptor, constitutes one of the principal monocyte/macrophage chemokines [6, 7]. Moreover, the concentration of CCL2 correlates positively with the stage of the tumor in cancer of urinary bladder and breast. High level of this cytokine is associated with poor prognosis; it is observed in patients with higher clinical stages of the tumor [8, 9]. In contrast, a proportional decrease in the concentration of CCL2 is observed with increasing stages of gastric malignancies, both in the serum and in neoplastic tissue. Perhaps, this phenomenon results from enhanced “consumption” of MCP-1 and concurrent unchanged level of synthesis [10]. Interestingly, MCP-1 can be released also by macrophages located in the region of neoplastic growth [11]. This suggests direct involvement of these cells in the recruitment of peripheral monocytes. It should be noted, however, that the impaired expression of CCR2 receptor on the surface of tumor-associated macrophages leads to the reversal of the effect exerted on these cells by the discussed chemokine. Plausibly, this is one of the mechanisms enabling the maintenance of rich macrophage infiltrate in the region of neoplastic growth [12]. Expression of MCP-1 correlates positively with the level of vascular endothelial growth factor (VEGF), TNF-*α*, and IL-8, which suggests its involvement in the processes of neovascularization [11]. The migration of peripheral monocytes to the site of neoplastic growth can be also induced by monocyte chemotactic protein-2 (MCP-2/CCL-8) and monocyte chemotactic protein-3 (MCP-3/CCL-7). These cytokines show both a structural similarity to MCP-1 and an affinity to the CCR2 receptor [13]. The chemotaxis of monocytes is also induced by CCL5 (RANTES), whose level of expression correlates with the degree of macrophage infiltration and lymph node metastases of neoplastic cells [14]. RANTES and CCL2 stimulate monocytes to secrete metalloproteinase-9 (MMP-9) and metalloproteinase-19 (MMP-19), which suggests indirect involvement of the discussed chemokines in the degradation of basal membrane, and hence in the process of neovascularization [15, 16]. Furthermore, high concentration of CCL5 increases the probability of metastases in patients with gastric malignancies [17]. Also, factors released by the neoplastic cells, such as VEGF, IL-8, and angiopoietin-2 (Ang-2), exert chemotactic effect on monocytes/macrophages; additionally, they are involved in the processes of angiogenesis.

## 3. Regulation of the Process of Neovascularization

Angiogenesis, although physiologically necessary, underlies a number of diseases. Formation of new blood vessels is critical for neoplastic growth and results from the predominance of proangiogenic factors over those inhibiting angiogenesis [18]. Enhanced angiogenesis can be observed in very early stages of malignant growth [19, 20]. Molecules such as (VEGF), interleukin-8 (IL-8/CXCL8), basic fibroblast growth factor (bFGF), angiopoietin-1 (Ang-1), and angiopoietin-2 (Ang-2) are the main mediators of neovascularization released by the neoplastic cells, including the cells of gastric malignancies.

VEGF is synthesized and secreted by many types of neoplasms [21–23] and although high levels of this molecule can be observed both in the plasma and in the serum of patients, the serum concentration of VEGF is higher. This results from the secretory activity of thrombocytes, which release high amounts of this factor during the coagulation of blood [17]. The production of VEGF in human macrophages is regulated by NF*κ*B [24]. VEGF acts in the receptor-dependent manner [25], inducing the chemotaxis of peripheral monocytes as a result of interacting with VEGF-R1 [26]. In response to VEGF, activated monocytes/macrophages synthesize molecules, for example, metalloproteinase-9 [27, 28], which, as previously mentioned, is involved in the processes of angiogenesis. Although tumor-associated macrophages (TAM's) constitute the principal source of MMP9 in the zone of neoplastic growth, it should be noted that this molecule may be also synthesized by neoplastic cells, stromal neutrophils, fibroblasts, and mastocytes [29]. Moreover, the activity of VEGF leads to an increased permeability of blood vessels within the neoplastic tissue [30], proliferation of vascular endothelial cells [31], and the inhibition of dendritic cell maturation [32]. The majority of these processes are associated with the activation of VEGF-R2 receptor [33]. Therefore, the VEGF/VEGF-R2 system is connected with the initiation of neovascularization processes.

Similarly to VEGF, interleukin-8 is a chemokine with pro-angiogenic activity. High expression levels of this molecule have been observed in various types of neoplasms, directly correlated with the vascularization of proliferating tissue and poor prognosis, being the highest in the advanced stages of tumor development. Gastric cancer cells also show the expression of A (CXCR1) and B (CXCR2) receptors for IL-8. During *in vitro* IL-8 stimulation, they show increased expression of epidermal growth factor receptor (EGFR), MMP-9, VEGF, and IL-8 [34]. Similarly to VEGF, interleukin-8 induces the migration of monocytes/macrophages to the site of neoplastic growth.

Basic fibroblast growth factor (bFGF) is one of the strongest stimulators of angiogenesis [35], acting via FGF-R1 and FGF-R2 receptors. Its expression is observed in many types of neoplasms [36]. High level of bFGF correlates positively with poor prognosis, and its expression in neoplastic cells is associated with the vascularization of the tumor. Moreover, this factor stimulates the chemotaxis of macrophages [37], which acquire the potential to synthesize and secrete this molecule in response to mediators released by neoplastic cells [38]. This suggests indirect TAMs-dependent influence of bFGF on the processes of tumor neovascularization.

Angiopoetin-1 (Ang-1) and angiopoetin-2 (Ang-2) are the main representatives of angiopoietin family. The activity of Ang-2 during the development of neoplastic disease is associated with the progression of the disease and the neovascularization of the tumor [39]. Angiopoietin-1 activates Tie-2 receptor (angiopoietin receptor), in this way stimulating *in vitro* migration of endothelial cells; moreover, it recruits pericytes into the newly formed vessels in order to stabilize their structure. In contrast, angiopoietin-2 is a natural antagonist of Tie-2 receptor. Ang-2 inhibits the maturation of vessels resulting from Ang-1 activity in a VEGF-independent manner and causes their regression. Therefore, it plays a regulatory function [34]. The activity of Ang-2 leads to the destabilization of vessels, which is necessary for the initiation of neovascularization process [40]. Angiopoietin-2 exerts positive effects on the processes of angiogenesis through VEGF involvement [34]. Interestingly, VEGF causes an increase in Ang-1 expression, but it does not modulate the synthesis of Ang-2 [41]. The level of Ang-2 expression correlates significantly with the clinical stage of disease (lymph node and organ metastases) while the expression of Ang-1 is significantly higher in poorly differentiated tumors [40]. The angiopoietin/Tie-2 system is involved in the remodeling and maturation of blood vessels and is, therefore, complementary to the activity of VEGF [34]. Moreover, the evaluation of Ang-1, Ang-2, and Tie-2 serum concentrations seems to be useful in preoperative differentiation of malignant thyroid tumors [42]. Additionally, angiopoietins are able to stimulate the chemotaxis of Tie-2-positive peripheral monocytes, which, constituting cells with pro-angiogenic potential, support the proliferation of neoplastic tissue [43].

## 4. Immunosuppressive Effect

Cancer cells and tumor-infiltrating leukocytes (primarily macrophages) modulate the activity of the immune system, also by means of immunosuppression, *via* the profile of released factors (cytokines and chemokines). IL-10 and TGF-*β* are released by these cells and show an array of immunosuppressive effects, for example:

- blockade of the activity of cytotoxic NK cells [44], macrophages, and cytotoxic T lymphocytes (CD8^+^) [45];
- reduced expression of class II MHC molecule on the surface of antigen presenting cells [46];
- polarization of immune response towards Th2 [47];
- inhibition of dendritic cell maturation [48];
- inhibition of certain functions of T lymphocytes [49];
- stimulation of tumor cell B7-H3 expression [50].

Together with a strong anti-inflammatory signal, neoplastic cell-released chemotactic factors for monocytes and macrophages induce their differentiation into MII macrophages, and hence into cells showing an array of functions promoting the proliferation of neoplastic tissue. Consequently, it should be noted that aside from direct immunosuppressive activity, growing neoplasm induces anti-inflammatory activity of infiltrating cells, thus escaping from the control of the immune system.

**Figure 1:**
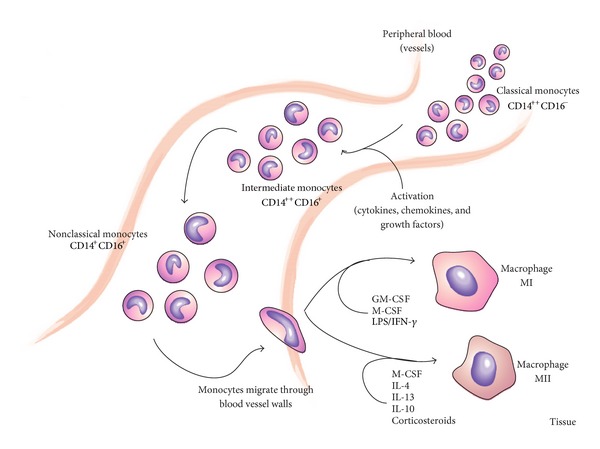
Differentiation of monocytes towards macrophages.

Macrophages are terminally differentiated cells of bone marrow origin that reside in tissues and are derived from peripheral monocytes (Figure 1). Depending on the activating factor, monocytes and macrophages can be involved in an array of biological processes, such as:

- presentation of antigen;
- cytotoxicity;
- phagocytosis;
- secretion of biologically active molecules;
- control of inflammatory processes;
- rearrangement and reconstruction of destroyed tissues [51].

As previously mentioned, the proliferation of neoplastic tissue modulates the activity of immune cells, including the function of monocytes and macrophages. It is widely known that macrophages residing at the site of neoplastic growth, referred to as TAMs, constitute the predominant component of infiltrate in many neoplasms, including gastric malignancies [52–54]. Due to their pleotropic biological properties, TAMs can have both progressive and regressive effects on the development of neoplastic tissue. Moreover, they control primary and secondary immune responses. The pro- or anti-neoplastic activity of macrophages is directly associated with their pro- or anti-inflammatory activity, respectively, and tightly depends upon monocyte-activating factors, which define the relevant polarization of these cells (Figure 1).

## 5. Activity of Monocytes

As peripheral cells, monocytes do not have direct contact with a neoplastic tissue. Indirectly, however, they are subject to its immunomodulatory effect, responding to chemotactic factors and neoplastic antigens present in peripheral blood, as well as to circulating neoplastic cells. All these factors directly induce the differentiation of monocytes to macrophages and their polarization towards pro- or anti-inflammatory cells. In the case of some malignancies, such as colorectal cancer, elevated monocyte count is considered as an independent prognostic factor [55].

Monocytes are heterogeneous population of cells in terms of morphology, phenotype, and effector properties. On the basis of the level of expression of lipopolysaccharide (LPS) receptor (CD14) and Fc*γ* receptor III (CD16), they can be classified into three well-characterized subpopulations

- those showing strong expression of CD14 receptor and lacking the expression of CD16 receptor (CD14^++^CD16^−^);
- those showing strong expression of CD14 and the presence of CD16 receptor (CD14^++^CD16^+^);
- those showing weaker expression of CD16 receptor than in the above-mentioned groups expression of CD14 receptor, with simultaneous (CD14^+^CD16^+^) [56].

Monocytes with CD14^++^CD16^−^ phenotype are referred to as classical monocytes and correspond to 85–95% of all peripheral monocytes under physiological conditions. The remaining two populations showing strong expression of CD16 receptor differ from each other in terms of CD14 expression level and, under physiological conditions, represent up to 15% of peripheral monocytes [57, 58]. Despite phenotypic similarities associated with the expression of CD16 receptor, stronger, as compared to classical monocytes, expression of HLA-DR, CD86, and CD54, and lower level of CD64 expression, both of the aforementioned subpopulations of monocytes show different biological activities [59]. An increase in the fraction of CD14^+^CD16^+^subpopulation of peripheral monocytes was observed during infections and inflammatory processes [60, 61], in septic states [62], and in some types of neoplasms [63, 64]. CD14^+^CD16^+^ cells are referred to as proinflammatory monocytes, because in contrast to classical monocytes upon stimulation they synthesize and release high amounts of tumor necrosis factor-alpha (TNF-*α*) without concomitant secretion of IL-10 or with low secretion of this cytokine [65]. The cells from this population show an array of similarities to tissue macrophages, and they are, therefore, postulated to be more mature and macrophage-like cells than the classical monocytes [66, 67]. Higher fraction of monocytes from CD14^++^CD16^+^ subpopulation has been observed in a number of conditions, including septic neonatal states [68] and gastric malignancies [69]. Additionally, compared to the pro-inflammatory subpopulation and classical monocytes, these cells show a higher expression of CD11b and TLR4 [59]. Moreover, they differ from the CD14^+^CD16^+^ subpopulation in terms of characteristics such as higher phagocytic activity [70] and the presence of anti-inflammatory properties, constituting the principal source of IL-10 amongst peripheral monocytes. The presence of this subpopulation in peripheral blood is thought to constitute an intermediate stage in the differentiation of monocytes to macrophages [59].

In spite of the lack of direct contact with neoplastic tissue, peripheral monocytes represent an interesting object during the assessment of the developmental stage of the disease. As previously mentioned, they undergo a continuous stimulation by chemokines and cytokines released by neoplastic cells and tumor-infiltrating cells, as well as by neoplastic antigens and circulating neoplastic cells.

CD14^+^CD16^+^ cells are the main subpopulation of monocytes showing *in vitro* antineoplastic activity, which is directly associated with the enhanced synthesis and secretion of cytokines such as TNF-*α*, IL-12p40, and IL-12p70, lack of synthesis and release of IL-10, enhanced synthesis of reactive nitrogen species, and higher cytotoxic and cytostatic activities [71]. Obviously, IL-12p40 and IL-12p70 do not exert direct anti-neoplastic effect. The influence of IL-12p70 is associated with the activation of IFN-*γ* synthesis in lymphocytes, which in turn contributes to the polarization of immune response towards Th1, that is, a proinflammatory response against neoplastic cells. Additionally, IL-12p70 activates cytotoxic T lymphocytes (CD3^+^CD8^+^) and NK cells, both showing an array of antineoplastic properties [72]. IL-12p40 is a chemotactic factor for monocytes, which differentiate into macrophages and migrate to the tissue. Therefore, IL-12p40 enhances the infiltration of macrophages into the site of neoplastic proliferation [73], where, as pro-inflammatory cells, they can exert many antineoplastic effects. The results of *in vitro* studies suggest that increased fraction of CD16^+^ monocytes in patients with malignancies can represent a natural consequence of immune response against neoplastic tissue as well as against circulating neoplastic cells. Spontaneous increase in this population of peripheral monocytes was also observed *in vivo*, including gastrointestinal malignancies [64].

Additionally, a decrease in the fraction of the subpopulation 1 of T-helper lymphocytes (Th1) was observed in peripheral blood of cancer patients in relation to the subpopulation 2 (Th2); this was associated with lower plasma concentration of molecules such as IL-2 and IFN-*γ*, and higher level of cytokines, such as IL-4, IL-10, and IL-13, as compared to healthy individuals [74]. In view of the elevated concentration of anti-inflammatory factors, circulating monocytes should gain anti-inflammatory properties and differentiate into MII macrophages, which are involved in the processes of neovascularization, among others. Proangiogenic activity is also characteristic for monocytes that show the surface expression of angiopoietin receptor (Tie-2), although this receptor is mostly expressed on epithelial cells and is considered as a specific feature of vascular epithelial cells [75]. Tie-2^+^ monocytes represent a separate population of cells referred to as the Tie-2-expressing monocytes (TEMs). Even though their physiological fraction in peripheral blood is low and corresponds to only 1-2% of peripheral leukocytes, approximately 20% of circulating monocytes are Tie-2^+^ [43, 76]. An increase in the fraction of Tie-2-expressing monocytes, even up to 10% of all peripheral blood mononuclear cells (PBMCs), has been observed in cancer patients. Murine model studies revealed strong pro-angiogenic properties of TEMs during the processes of neoplastic tissue neovascularization. Moreover, these cells are considered the precursors of pro-angiogenic tissue macrophages [76], which correspond to up to 30% of all TAMs in certain parts of the neoplastic tissue [77].

## 6. Macrophage Activity

Macrophages are the predominant cells of the leukocytic infiltrate of many neoplasms and are able to polarize their immune response in both a pro- or anti-inflammatory direction. Monocytes, activated by microorganisms or their parts, certain pro-inflammatory cytokines (e.g., IFN-*γ*), GM-CSF, and M-CSF, migrate into tissues and differentiate into pro-inflammatory cells, referred to as MI macrophages, which are involved in the destruction of microorganisms and neoplastic cells, among others (Table 1). Their function includes the activation of immune system and the support of adaptive response by means of:

- enhanced synthesis and secretion of pro-inflammatory cytokines such as TNF-*α*, IL-1, IL-6, IL-12, and IL-23 [78–80];
- enhanced synthesis and secretion of chemokines such as CCL5, CCL8, CXCL2, and CXCL4 [81–83];
- polarization of immune response towards Th1 and/or Th17 [84];
- high capacity for presentation of antigen to antigen-naive T lymphocytes;
- cytotoxic potential [85].

Monocytes activated by factors such as IL-4, IL-13, IL-10, and M-CSF show typical activities of anti-inflammatory cells; when present in tissues, they are referred to as MII macrophages (Table 1). These cells are characterized by:

- enhanced synthesis and secretion of anti-inflammatory cytokines such as IL-10, TGF-*β*, and IL-1RA;
- enhanced synthesis and secretion of chemokines such as CCL16, CCL18, and CCL22;
- polarization of immune response towards Th2;
- induction of T-regulatory (Treg) lymphocyte formation;
- low capacity for the presentation of antigen to antigen-naive T lymphocytes;
- strong expression of arginase-1 (its activity alters the metabolism of L-arginine into ornithine and polyamines, which results in the blockade of inducible nitric oxide synthase (iNOS));
- lack of cytotoxic activity;
- higher expression of certain membrane receptors, including type 2 Fc receptor for IgG (Fc-R2, CD23), mannose receptor (MR), and receptor for LPS (CD14) [85].

The principal tasks of MII include the suppression of adaptive response, inhibition of cytotoxic cell activity, rearrangement and reconstruction of destroyed tissues, and their neovascularization. Therefore, MII macrophages play a regulatory function in pro-inflammatory response *via* the control of MI cell-dependent activities [86]. Consequently, the maintenance of body homeostasis requires maintaining a proper ratio of both discussed subpopulations of macrophages, namely, MI and MII. This balance is disturbed during such pathological conditions as the proliferation of neoplastic cells, leading to the impaired activity of immune system and uncontrolled progression of the disease. This is unambiguously associated with the immunomodulatory effect of proliferating neoplasm. However, the secretory activity of macrophages residing within the tumor should not be forgotten. The activity of macrophages in the course of neoplastic disease has been studied extensively as they can exert both progressive (MII macrophages) and regressive (MI macrophages) effects on the development of neoplastic tissue. The balance between MI and MII macrophages seems to be controlled by NF*κ*B signaling, because targeting of this transcription factor switched macrophages from an MII to an MI phenotype. The consequence of which is the regression of tumor tissue *in vitro* [87].

As pro-inflammatory cells, macrophages are involved in stromal remodeling releasing a slate of pro-inflammatory factors that constitute a signal of danger for immune cells. This macrophage activity may result in the following:

- activation of cytotoxic T lymphocytes and NK cells;
- influx of dendritic cells;
- migration and differentiation of monocytes in a pro-inflammatory direction.

Activated MI macrophages synthesize and release IL-12, which, as was mentioned previously, shows indirect antineoplastic activity. Additionally, it exerts stimulatory effect on NK cells, inducing the synthesis and secretion of IFN-*γ* [88] and enhancing their cytotoxic potential. Stimulated with this cytokine, macrophages release factors such as ROI, IL-1, IL-6, arginase, and TNF-*α*, that is, pro-inflammatory factors exerting cytostatic and cytotoxic effects upon neoplastic cells. Moreover, these cells show a strong cytotoxic activity in both the antibody-dependent (ADCC) and antibody-independent (MTC) mechanisms [89].

Unfortunately, the majority of macrophages infiltrating neoplastic tissue have phenotype characteristic for MII [85], and thus they present an array of activities promoting the growth of neoplastic tissue. Consequently, tumor-associated macrophages are considered to constitute MII-like cells. The level of their infiltration is used as an independent prognostic factor in many tumor types. However, it should be noted that in the case of some malignancies, for example, colorectal and gastric cancers, higher fraction of these cells does not necessarily correlate negatively with patients' survival. The activity of TAMs in gastric malignancies can vary depending on tumor region. For example, higher infiltration of TAMs to the region of tumor cell nests is associated with improved survival. Despite the small fraction of nest TAMs, as compared to their overall count in the other regions of the tumor, enhanced apoptosis of neoplastic cells was observed along with a higher activity of cytotoxic T lymphocytes. Consequently, it should be emphasized that antineoplastic activities controlled by macrophages with MI phenotype can be induced in certain regions of neoplastic tissue [52]. However, they represent a minority of pro-neoplastic activities of MII macrophages, associated with the following:

- suppression of adaptive response;
- promotion of tumor growth;
- promotion of the metastases of neoplastic cells;
- involvement in the recruitment of peripheral monocytes and macrophages from the surrounding tissues.

The MII macrophages release an array of anti-inflammatory factors, such as IL-10 and prostanoids, causing the attenuation of type Th1 immune response, as well as an impaired activity of cytotoxic T lymphocytes and NK cells. Moreover, they secrete a variety of specific cytokines (e.g., CCL17 and CCL22), inducing the inflow of regulatory T cells and Th2 subpopulation of helper lymphocytes. These effects are reflected by the suppression of pro-inflammatory activities of the immune system, that is, by the inhibition of activities oriented against neoplastic cells [86]. Moreover, tumor-associated macrophages are capable of modifying *ζ* subunit of TCR receptor (TCR-*ζ*) of T-helper lymphocytes [90–92], which plays a crucial role in the activation of the latter cells [93]. The disorders of TCR-*ζ* expression or inactivation of this subunit are reflected by the anergy of T lymphocytes, leading to their apoptosis.

Intensified proliferation of neoplastic tissue is associated with an increased requirement for nutrients and growth factors and leads to the hypoxia of the tumor. High efficiency of pro-neoplastic MII macrophage activity is associated with their ability to accumulate within the oxygen-deficient regions of the tissue. TAMs synthesize and release an array of growth and pro-angiogenic factors that are concurrently chemotactic factors for monocytes and macrophages, including VEGF, bFGF, CXCL8, PDGF, EGF, and TGF-*β* [89, 94]. PDGF promotes the proliferation of neoplastic tissue; additionally, it acts as a pro-angiogenic factor and recruits pericytes stabilizing the newly formed vessels [95]. EGF stimulates neoplastic cells to synthesize and release M-CSF. Aside from the chemotaxis of macrophages from the surrounding tissues, M-CSF induces the differentiation and migration of peripheral monocytes. This constitutes one of the mechanisms behind the enhanced infiltration of macrophages into the tumor, which is in turn reflected by an enhanced synthesis of EGF. EGF/M-CSF feedback cycle leads to macrophage-dependent, growth factor-induced intensive proliferation of neoplastic tissue [96].

Macrophages present at the invasive front of the tumor (margin TAMs) participate in the creation of promoting environment for neoplastic cells, enabling them to reach vascular and lymphatic system. As previously mentioned, TAMs constitute a source of metalloproteinases (such as MMP-2 and MMP-9) and an urokinase-type plasminogen activator (uPA) [97], which facilitate tumor invasion due to their involvement in the degradation of basal membrane and extracellular matrix. This process seems to a large extent to be EGF and M-CSF dependent. The blockade of EGF and M-CSF activities is reflected by inhibited migration of neoplastic cells and macrophages, respectively [98, 99]. Therefore, the M-CSF-stimulated TAMs induce the migration of neoplastic cells on EGF-dependent pathway [100]. Most likely, this process is also modulated by IL-4, which polarizes macrophages towards promoting the invasiveness and spread of the tumor; these functions are blocked in the lack of IL-4 [101, 102]. The invasiveness of neoplastic cells can be also modulated in TNF-*α*-dependent manner [103], with macrophages being the principal source of TNF-*α* [104, 105]. The migration of neoplastic cells through the stroma is markedly more efficient if supported by collagen fibers (type I collagen) [106], formed with the involvement of TAMs. These fibers expand towards blood vessels [107], significantly facilitating the migration of neoplastic cells and thus promoting the formation of distant metastases. In summary, due to their pro-angiogenic activity, synthesis of collagen fibers, and the induction of neoplastic cell migration, TAMs actively promote the invasion and spread of the tumor.

## 7. Conclusion

The process of neoplastic tissue proliferation is directly related to the modulatory effect on the immune system. Monocytes/macrophages are particularly susceptible to this effect. This results in a switch from a protective function into one that promotes neoplastic proliferation. In the case of many malignancies (e.g., breast, prostate, or endometrial cancer), high percentage of TAMs is associated with poor prognosis. In practice, high percentage of tumor-associated macrophages may be an independent prognostic factor, providing a thorough examination of all the regions of the tumor and a precise assessment of the phenotype of these cells. TAMs also seem to be a promising target for antineoplastic therapy, the aim of which should be, for example, the reversal of the unfavorable balance between MI and MII macrophages [87]. On the other hand, monocytes/macrophages can be used as a delivery system of anticancer agents to tumors [108].

## Figures and Tables

**Table 1:** Macrophage subsets.

Populations	Inducing agents	Functions
	GM-CSF; IFN-*γ* + LPS; TNF-*α*	(i) High capacity for antigen presentation
		(ii) Th1 polarization
MI		(iii) Defense against bacteria
		(iv) Tumor suppression
		(v) Immunostimulation
		(vi) Ability to induce a cytotoxic effect

MII		
MIIa	IL-4; IL-13	(i) Th2 polarization
MIIb	Immune complex;	(ii) Down-regulation of adaptive immunity
	IL-1R agonists; TLR ligands	(iii) Tumor growth promotion
MIIc	IL-10; TGF-*β*;	(iv) Proangiogenic
	glucocorticoids	(v) Tissue remodeling and repair
